# A pre-registered naturalistic observation of within domain mental fatigue and domain-general depletion of self-control

**DOI:** 10.1371/journal.pone.0182980

**Published:** 2017-09-20

**Authors:** Daniel Randles, Iain Harlow, Michael Inzlicht

**Affiliations:** 1 Department of Psychology, the University of Toronto, Toronto, Ontario, Canada; 2 Learning and Data Science group, Cerego, San Francisco, California, United States of America; 3 Rotman School of Management, the University of Toronto, Toronto, Ontario, Canada; Mälardalen University, SWEDEN

## Abstract

Self-control is often believed to operate as if it were a finite, domain-general resource. However, recent attempts to demonstrate this under transparent conditions have failed to yield positive results. In the current study, we monitor two groups of students (N1 = 8,867, N2 = 8,754) over separate 17-week intervals with 24-hour coverage, as they engage in voluntary learning and self-testing using an online program. We use daily behavior to assess whether time-of-day effects support domain-general theories of self-control. Additionally, we assess whether mental fatigue emerges within task during prolonged persistent effort. Results reveal within-task fatigue emerges within an hour on-task. However, there is a negligible effect on ability throughout the day. Additionally, time-of-day has no detrimental effect on motivation; rather there is a strong tendency to increase learning time at night. Results are consistent with theories indicating people lose motivation within a specific task, but at odds with theories that argue for a domain-general self-control resource.

## Introduction

Is self-control limited? If we spend our day focused on work, being careful about food choices, and polite to our coworkers, are we doomed to spend evenings eating chips in front of the television? Many people would say yes, that using self-control is exhausting, leaving them vulnerable to unproductive short-term desires. Until recently, many psychologists would also endorse this belief, saying that self-control operates as if it draws on a central, domain-general resource. However, recent empirical and theoretical challenges have made this common wisdom more uncertain than previously thought. In particular, two questions have emerged. First, does engaging self-control in one task influence an individual’s ability to use self-control in unrelated tasks? Second, if self-control in fact can become depleted within any one task, how long does it take before a noticeable drop in performance is evident? Considering these issues, it is important to demonstrate that domain-general depletion of self-control can be observed in natural settings, as a step towards validating its existence and time course.

Until recently, laboratory work in psychology has consistently supported resource models of self-control [1–3], which claim that self-control relies on a finite, domain-general resource, and is needed to direct behavior away from one’s automatic preferences or impulses. When active control in one-domain depletes an individual (e.g. attending course lectures), this should lead to a state of weakened self-control in any other domain (e.g. controlling how much one drinks at a party); a state often described as ego depletion. To date over 200 studies have demonstrated that engaging in a task that requires impulse control or mental effort leads to inhibition failures in an unrelated subsequent task [4], [5].

However, recent attempts to replicate the domain-general aspect of self-control under transparent research conditions have failed to produce positive results [6–7]. The null-results of these pre-registered efforts mirror meta-analytic work, suggesting that despite the hundreds of published experiments, the underlying effect of depletion has been over-estimated, if it exists at all [5, 8]. These recent contentious findings have challenged one of the core assumptions of the resource model of self-control: That engaging self-control leads to a spillover effect, whereby self-control is limited for any unrelated task.

In contrast, evidence of motivation and performance declines when persisting on a single, prolonged task, is on much firmer ground. Large, longitudinal assessments of within-task mental fatigue consistently show an effect over a period of hours. For example, in an analysis of 2 million grade school tests over 3 years, researchers in Denmark found a consistent decline in student scores over the course of the day, but that taking breaks could potentially eliminate this decline completely [9]. The rejuvenating effect of breaks is important, as it builds a stronger case that the lower scores are at least somewhat independent from other time-of-day effects, such as changes caused by circadian rhythms. Likewise, eight judges presiding over roughly 1,100 parole hearings were found to grant parole most often at the beginning of their workday, though as with the Danish students, break periods and food reversed this trend almost to a return to baseline levels [10]. In both of these cases, it could be argued that the samples provide clear evidence for within-task fatigue; persistent effort applied to one task (schoolwork, judgments) alters a person’s ability to perform consistently at that task. However, these studies do not clearly speak to the idea that persistent effort in one domain decreases self-control and motivation in other domains. That is, after a day of work, would an individual be less inclined to adhere to a non-work related commitment because of their general depletion? Additionally, while these studies speak to reduced ability and compliance over time, they cannot speak to willingness to voluntarily engage in a strenuous task after having been taxed by other stressors. The data we present below address these gaps, by presenting a large naturalistic observation of voluntary learning with 24-hour coverage.

Our research was completed in collaboration with Cerego, an education software company. Amongst other services, Cerego offers online supplemental study packages that accompany courses and textbook material. From this data, we can assess when users are most likely to log on, how long they persist at a session, and how successful they are at memory tests. We consider three questions that previous studies have been unable to address.

First, do users show altered ability to engage in learning as a function of the time of day? Assuming most people are required to use self-control throughout the day, daily requirements to engage effortful control should lead to a domain-general depletion effect, reducing voluntary learning later in the day. Were this effect to emerge, it could be caused by reduced motivation to learn, or by reduced ability to inhibit desires to engage in other more subjectively-satisfying activities. Untangling these motivations is beyond the scope of our data, but would provide evidence for a world that was aligned with resource models of self-control.

Second, regardless of explicit motivation, do users show altered ability to correctly remember information as a function of the time of day? That is, even if they are willing to log on and complete sessions later in the day, are they less able to marshal the cognitive resources to succeed at the task. This is the most comparable test of ego-depletion to most laboratory studies. Under the assumption that users want to do well when they voluntarily choose to learn, reduced performance would be analogous to increasing errors on a laboratory cognitive task.

Third, what is the magnitude of fatigue caused by persistent effort within the task, and how does it compare to time-of-day effects? In other words, do users show signs of fatigue and reduced ability as they persist within a single session, and if so, is that effect larger or smaller than the hypothesized effect of domain-general self-control depletion. This question addresses whether within-domain fatigue is present in our task, and allows us to compare the effects of persistently applied effort within a single domain to the effect of domain-general depletion emerging over the course of the day.

## Materials and methods

**Fig 1:**
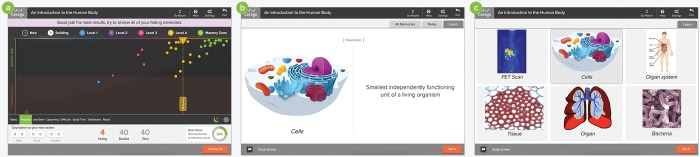
Example images from the website http://www.cerego.com. Example screens from a user’s session on Cerego. (A) When users log on, they see a visual summary of information they have learned on Cerego. (B) Learning trials involve paired information, in this case the name and visual representation of an osteoclast paired with its definition. (C) On a test trial, users will be asked to either freely recall information, or select correct options from a set. In this case the user must identify the correct name and representation after having been given the definition. Printed under a CC BY license, with permission from Cerego, original copyright 2017.

Cerego presents users with slides that contain a discrete piece of information on the topic of choice (Fig 1). For language learning, this might be matching a word in the learned language with a relevant image; for topic learning, a name and definition might be paired. Each trial presents one specific piece of information, and that information is logged as a “memory” in the user’s profile. Over time, the system presents learned information back to the user in the form of memory tests based on recall (free-response) and recognition (multiple choice). When a user answers an item correctly, the system delays the next exposure based on an algorithm, which anticipates when the user would be at risk of forgetting the information. In this way, Cerego minimizes how often users must repeatedly test themselves, and maximizes the likelihood that a test session will help to consolidate the information into long-term memory. Users receive email notifications when they have a sufficiently large number of memories that Cerego has identified as needing to be reviewed. Email notifications are based solely on the user's learning algorithm and study materials; they are not tied to a time of day.

Cerego provided data from two cohorts of students using their system (N1 = 5,666; N2 = 8,754) over two seventeen-week periods beginning September 2015 and January 2016. In sample 1, users contributed 72,082 learning sessions, with a mean of 12.82 sessions per user, SD = 11.40; sessions lasted an average of 61.21 trials or 17.73 minutes (SDs = 48.73, 13.47). For sample 2, 147,186 sessions were provided, mean per user = 16.81, SD = 15.71; sessions lasted a mean of 58.63 trials or 16.78 minutes (SDs = 45.32, 13.26). Users were enrolled in undergraduate level psychology courses that had purchased institutional access to Cerego. No demographic information was avaiable (e.g. age, gender, institution), however most users were likely college-age adults. Based on sample 2 time-zone data, over 99% of users were located within UTC -09 to UTC -05 (corresponding with Pacific to Eastern time in North America).

Our interpretation of the Cerego service is that it can be described as a mentally challenging task. Every session includes learning new information, as well as being tested on information stored in short (minutes to hours) and longer-term (days) memory. To observe mental-fatigue within task, we can assess how persistent effort within a specific session either reduces or improves an individual’s ability to answer questions correctly. To assess the effect of domain-general depletion, we built a 24-hour model of Cerego behavior, revealing time-of-day effects for how likely an individual is to log in, how long they will persist at the task, and whether they show improved performance at any point in the day.

Working under the assumption that our users engage in moderately mentally stressful activities throughout the day, the resource model of self-control would predict that users will be most willing to exert voluntary effort most in the morning and least in the evening. The assumption that the general population shows self-control depletion throughout the day is a fairly strong one. However, if resource models of self-control are correct, it is very difficult to generate a plausible scenario of daily behavior where this wouldn’t be the case. In S1 Text, we describe simulated environments that might reflect daily lives for a population. Even in cases where people often do not require self-control, and have many opportunities to rest, a population level decline of self-control emerges within an hour of wake-time and persists throughout the day (See Figure in S1 Text).

**Fig 2:**
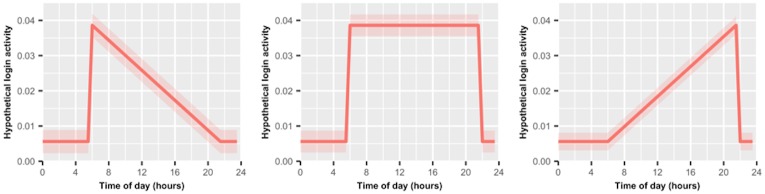
Theoretical models of user activity as a function of time of day. Models of user behavior based on different self-control theories. Solid line represents hypothesized average behavior for a sample throughout the day, Colored region is hypothesized standard deviation. (A) Assumes that a domain-general reserve is required for all self-control and requires rest to refill. (B) Assumes that self-control is stable throughout normal waking hours and has no limits. (C) Assumes self-control is stable throughout normal waking hours, and additionally that people have the most opportunity for personal projects later in the evening. Figures present parameters for login activity, each analysis is defined by its own parameters.

Sample 1 analysis was exploratory, after which we pre-registered an analysis plan (based on sample 1 results) and began confirmatory analysis on sample 2 (OSF pre-registration is available at http://tinyurl.com/zby5pzt). At the time of the registration, Cerego had collected sample 2 as part of their normal business operation, but had not provided data to our research team. We used several screening procedures to remove users or sessions that we identified as problematic (See S2 Text, pre-registration for details). Beyond fitting our models to the data, we additionally compared our data to three theoretical patterns of behavior, as defined in our pre-registration (Fig 2). The first model labeled “Limited self-control” is what we should expect if self-control is drawn from a central finite resource. Although everyone’s day will be different, in aggregate we expect people to engage in effortful tasks throughout the day, producing some decrease in willingness and ability to learn as they day progresses (See S1 Text). The 2nd model labeled “Unlimited self-control” is akin to a null model, assuming low willingness and ability during late-night hours but otherwise flat performance. The 3rd model is similar to the null model, but assumes that users’ daily responsibilities keep them from personal learning during the day, thus forcing them to rely more on evening hours. See S3 Text for criteria to construct each model.

In sample 1, we did not have access to specific time zone information, which meant that an unknown number of our participants were offset by as much as 4 hours. When running confirmatory analyses, we did not correct for time zone data in sample 2, so that it would be analogous to sample 1. However, we did adjust users’ time-zones in sample 2 when refitting a new model to the data. Because of this discrepancy between the samples, the model fit to Sample 2 data is likely a more accurate estimate of time-of-day effects. Institutional ethical approval was granted by the Social Sciences, Humanities, and Educational Research Ethics Board at the University of Toronto (ref: 33748). Secondary analysis of anonymized user data for research purposes is covered by the Cerego End User License Agreement.

## Results

### Trial accuracy within session

We first ask whether there is evidence for reduced ability as individuals persist through the task. Analyses were run as multilevel models with user-defined random intercepts and slopes (details below). We compared linear and quadratic model fits to the null model based on their Aikaike information criteria, which estimates out-of-sample deviance accounting for varying complexity across models [11]. While absolute AIC scores aren’t interpretable, differences of 2 or greater between models are generally considered meaningful support for one model over the other. Because of the complexity of the models, we do not provide out-of-sample deviance for sample 2 as we will for time-of-day effects.

Sessions were a mix of learning and testing trials. We removed all learning events, but maintained the trial order for testing events. Accuracy was averaged over every 5 trials to smooth the effect and address the gaps produced by missing learn trials. Our analysis treats all trials as equally difficult, however trial difficulty is neither equal nor random. Cerego uses a proprietary algorithm to assign test items most likely to be forgotten closer to the beginning of the session. While we are unable to provide the details of that algorithm, we note that any effects of fatigue are likely conservative, as trials are more likely to be easy for a user the longer into the session they go. We used two analysis approaches, regressing accuracy onto either trial order or elapsed time of the session. This was done to address the possibility that people spent different amounts of time on trials either earlier or later into their session. Trial order or length was grouped within user but not within session, effectively controlling for inter-individual variance but not variance within individuals across sessions. This is not the ideal model, as it assumes variance of the effect across sessions for a user is not meaningful. However, in exploratory analyses with sample 1, we found that many of the models would not converge without this assumption.

**Fig 3:**
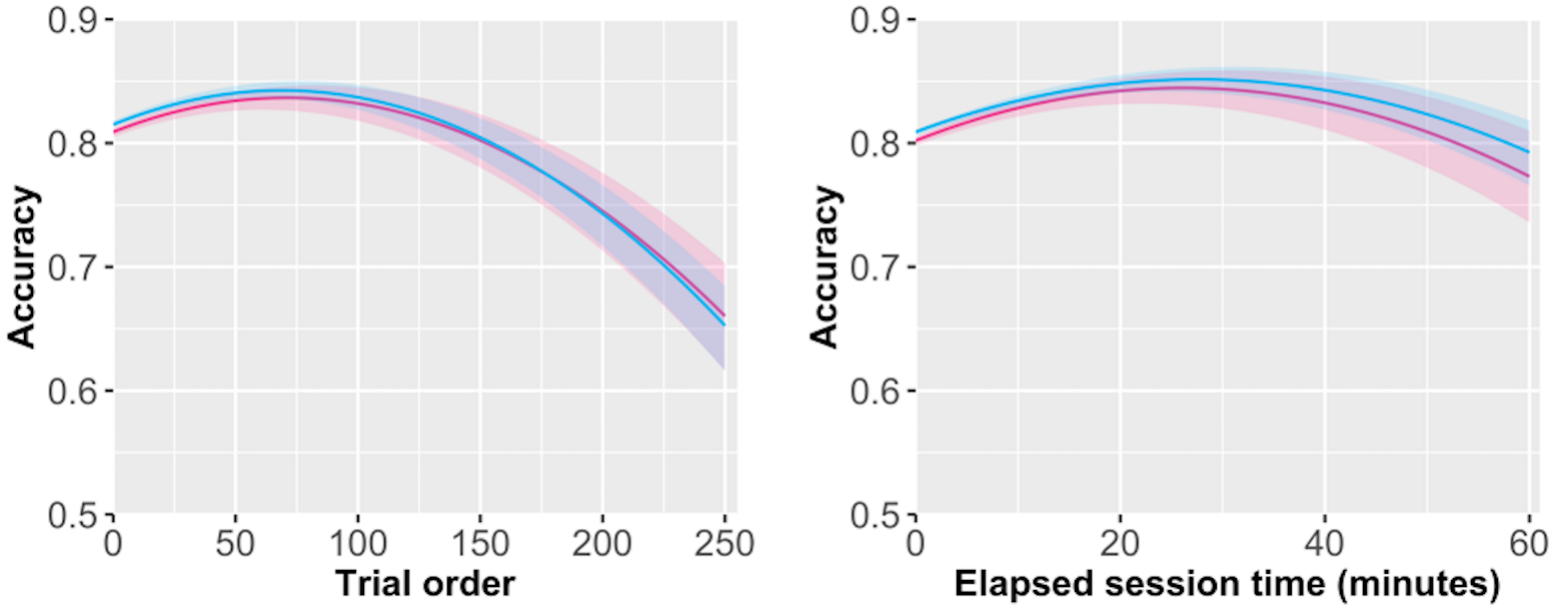
Quadratic regression of accuracy on trial length. Notes: Red = sample 1, Blue = sample 2. Average regression effect of (A) trial number and (B) elapsed time on accuracy. Shaded regions represent the 95% CI of the fixed effect.

In sample 1, we found that a quadratic function fit the data best relative to the other models, with an AIC score least 200 points lower, whether we defined trial progress based on the trial number or the amount of time elapsed; a finding that was replicated in sample 2 (Fig 3, S1 and S2 Tables for full quadratic models). User initially show an improvement in performance, then show a decrease starting by about trial 75 or 28 minutes, which leads to a 4–5% drop in performance from that peak by about trials 150 or 50 minutes into the session. Again, this is likely a conservative measure of user fatigue because of the non-random difficulty of test trials.

### Session accuracy throughout the day

Our first analysis identified that users experience a decrement in ability as they persist in the task. This section considers whether there is an effect of time-of-day on ability, indicating more domain-general depletion of ability that wasn’t directly caused by persistence on the task at hand. To assess this, we looked at the average score per session as a function of time-of-day. This involved finding the mean score for each session, and averaging all sessions within half-hour intervals across the day. Weighted linear regression was used to assess the relationship. In our analyses with sample 1, we found that many users provided either too few data points, or too little variance in terms of time-of-day, to run a MLM clustered within user.

We anticipated that if session accuracy had a measurable daily pattern, it would likely be sinusoidal, reflecting a daily pattern of increasing or decreasing ability, repeating itself each day. We tested sinusoidal regression lines from the first to fifth harmonic, as well as a linear, quadratic and intercept only (null effect) models. Harmonic functions are similar to exponential functions in that they allow the function to alter its acceleration to more precisely fit to the data. As with exponential transformations, harmonic transformations always improve the model fit at least slightly, and as such should be corrected for possible over-fitting. To account for this varying complexity across models, we used Watanabe-Akaike information criteria (or Widely-Applicable-Information-Criteria, WAIC) as the measure of model fit [11]. Models were fit with sample 1 and compared using WAIC. The exact models were then used to test out-of-sample deviance with sample 2 data based on root mean squared error (RMSE). This allows us to use the model from sample 1 and test how well it can predict new data relative to the other models. Finally, the models were re-fit with sample 2 data after adjusting for users’ time-zone.

**Fig 4:**
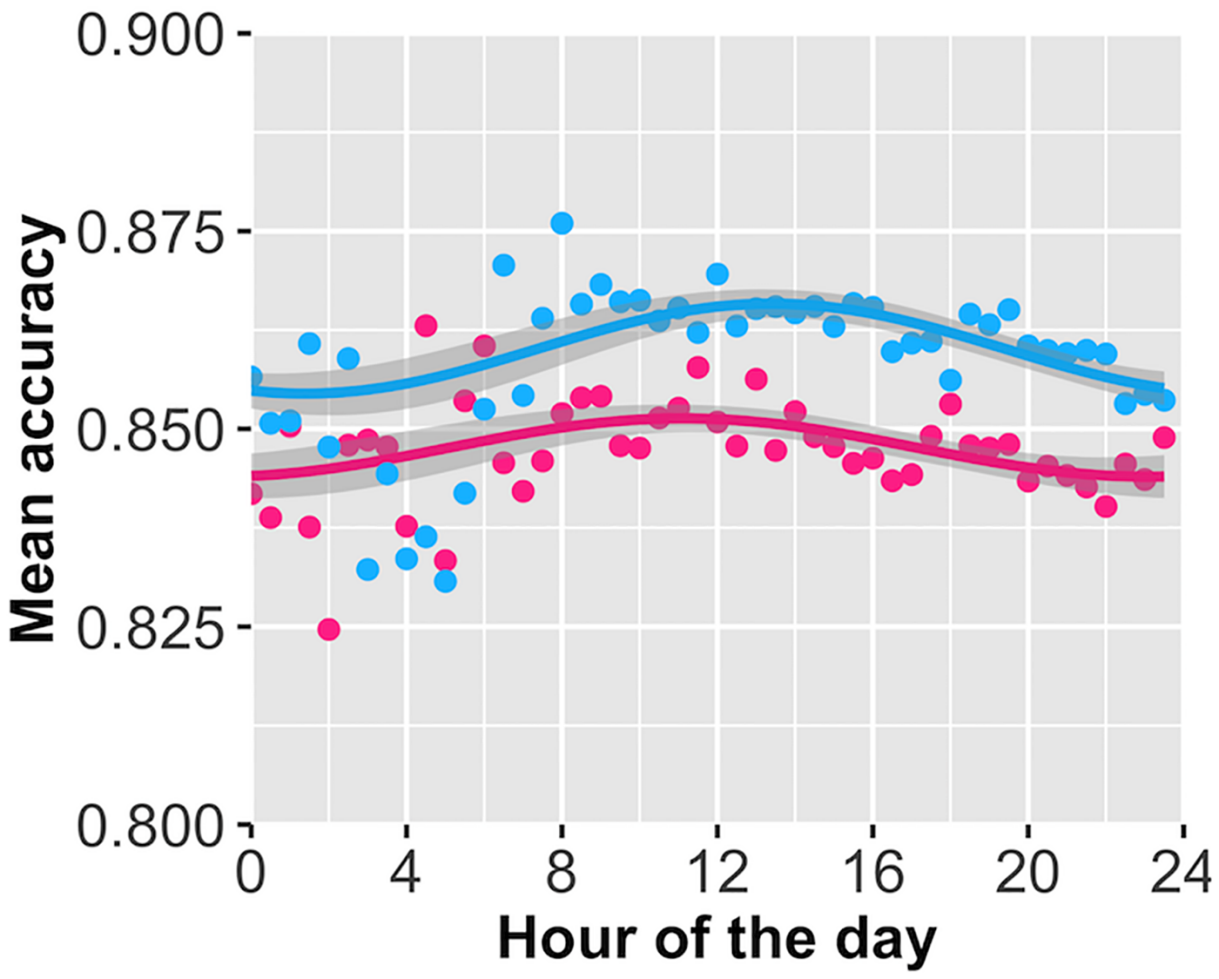
Daily session accuracy over 24 hours with 1st harmonic regression line. Notes: Both lines are 1^st^ harmonic regression lines fit to data. Red = sample 1 data. Blue = sample 2 adjusting for users’ time-zone. Shaded regions represent the 95% CI of the fixed effect. Dots represent the average score across sessions for each 30-minute window.

Our sample 1 results indicated that a 1st harmonic sine function was the best and most parsimonious fit for the data, with a WAIC score difference of at least 5.2 relative to all other models. RMSE from sample 2 largely supported this, though the small difference between all models highlight the small magnitude of the effect (RMSE ranged (2.03–2.08)*10^-4 across all models, the best model improved prediction by less than 4% against the intercept model). WAIC scores from the re-fit sample 2 data indicate that a 1st or 2nd harmonic function, or a quadratic model was the best fit. We focused on the 1^st^ harmonic function as it was the simplest model that was ideal for cyclical data (Fig 4, S3 Table For full 1^st^ harmonic regression models). This model indicates that performance drops throughout the day, albeit very slightly (roughly 1% drop in performance from peak to trough). While the effect is small, it consistently emerged in both samples.

Fitting to our theoretical models (Fig 2), the data in sample 1 were best fit to the model assuming limited self-control. However, the sample 2 data were no more likely under either the limited self-control or evening preference model, which predicts the exact opposite (Table 1). The failure of our models to clearly distinguish between a pattern of early morning or late evening ability in sample 2 comes from our initial expectation that peak performance occurred early in the morning, whereas our 2nd sample (once adjusted for time zone data) suggests the peak is closer to 2pm. This is important, because our initial peak was drawn from an expectation that self-control begins to deplete across the sample shortly after waking. That the peak emerges at 2pm is somewhat at odds with this. Given this failure to replicate our sample 1 findings, the data do not speak strongly in favor of a domain-general resource model of self-control. A coding error in our pre-registration led us to report that the unlimited self-control model was the best fit for data in sample 1.

**Table 1:** Deviance of accuracy scores relative to theoretical models for daily patterns of accuracy.

Model	Sample 1	Sample 2 direct replication	Sample 2 adjusting time zone
Limited SC	1.1*10^3^	8.84*10^6^	8.84*10^6^
Unlimited SC	2.8*10^3^	8.97*10^6^	8.97*10^6^
Evening preference	1.5*10^3^	8.84*10^6^	8.84*10^6^

Notes: SC = self-control. Values are the negative log likelihood of the data given the model. Smaller values imply less deviance and greater likelihood. See Fig 2 for representations of the theoretical models.

### Within-session fatigue effects moderated by time of day

Given that a small time-of-day effect did emerge for average session accuracy, we next considered whether this time-of-day effect moderated the specific components of the within-session fatigue effect. This is a departure from our pre-registered analysis and should be considered exploratory. The most straightforward approach would be to re-run the multilevel model assessing within-session fatigue, moderated by time-of-day. However, these models failed to converge for both samples. As an alternative approach, we attempted to fit multilevel models grouped within users and session. Although in general we struggled with convergence across previous attempts, we found that when using trial order as the independent variable, the quadratic model converged for both samples. These models produced similar results to our models that only group trials within user (for full models see S9 Table). This model provided us with an intercept, linear, and quadratic coefficient defining each session. We then regressed each of these terms on time-of-day, assessing whether a 1^st^ harmonic sinusoidal pattern emerged for any of the components. We reduced our critical p-value to .008 to control our error rate for the 6 tests (3 dependent variables being regressed onto the sine and cos function for time-of-day).

Of the three components, a moderation of the quadratic term would be most supportive of domain-general self-control, followed by the linear term. If the quadratic term was largest in the evening, it would be possible that other stressful activities throughout the day were increasing the within-session fatigue effect. Likewise, if the linear term were smaller later in the day, it would also suggest greater within-session fatigue in the evening, though less convincing, because the linear term was probably influenced more strongly by trials earlier in the session, as it served to connect the low intercept scores with better scores a few trials later. Finally, a moderation of the intercept by time-of-day would not offer support for self-control depletion, as it reflects how people responded to their first few questions.

**Fig 5:**
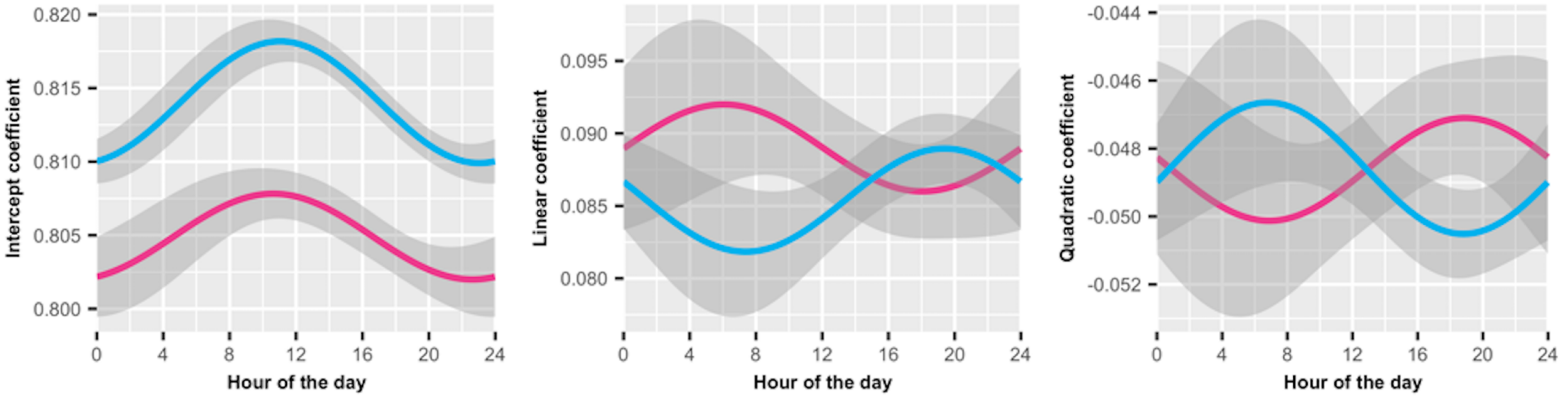
Time of day predicting the intercept, linear, and quadratic coefficients for within task fatigue. Notes: Sample 1 is red, Sample 2 is blue. Shaded regions represent 95% confidence intervals. Plots reveal that (A) there is a daily pattern for session score at the beginning of the session (Intercept term) but no significant or consistent pattern for either the (B) linear or (C) quadratic terms defining within-session fatigue.

We did not find a significant effect for either the linear (all *p* values over .03) or the quadratic term (all *p* values over .02). Critically, the relationship between these components and time-of-day was not consistent between the two samples, indicating this isn’t merely an issue of power (Fig 5; for full models see S6, S7 and S8 Tables). We did find a significant effect for time-of-day on the intercept term that was consistent across both samples and produced a similar diurnal pattern (Sample 1 sine(time) p = .25, cos(time) p = .003; Sample 2 sine(time) p = .17, cos(time) p < .0001; See Fig 5). This indicates that there was a certain time of day when users were most effective at answering their first few question in the set, which coincides with the hardest set questions. However, this is again a very small effect, influencing success on the initial session questions by less than 1%.

### Login activity based on time of day

In our first set of analyses, we found evidence that users perform worse the longer they persist at the task, suggesting that they find the task depleting. However, we find only a very small effect for time-of-day on session score, and in a follow-up moderation found that time-of-day was most relevant to the first few responses. While users appear no less successful at the task however, it is possible that they are nonetheless less motivated later in the day because of domain-general depletion. To assess the relationship between time-of-day and login activity, we summed up the number of users that began at any given time, in half-hour increments. We then rescaled the score for each half-hour window as a fraction of overall daily activity. This is a departure from our pre-registered analysis, where we initially used the raw sum for each window. The scale change was necessary as sample 2 turned out to contain substantially more sessions, making the two models inequivalent without scaling.

**Fig 6:**
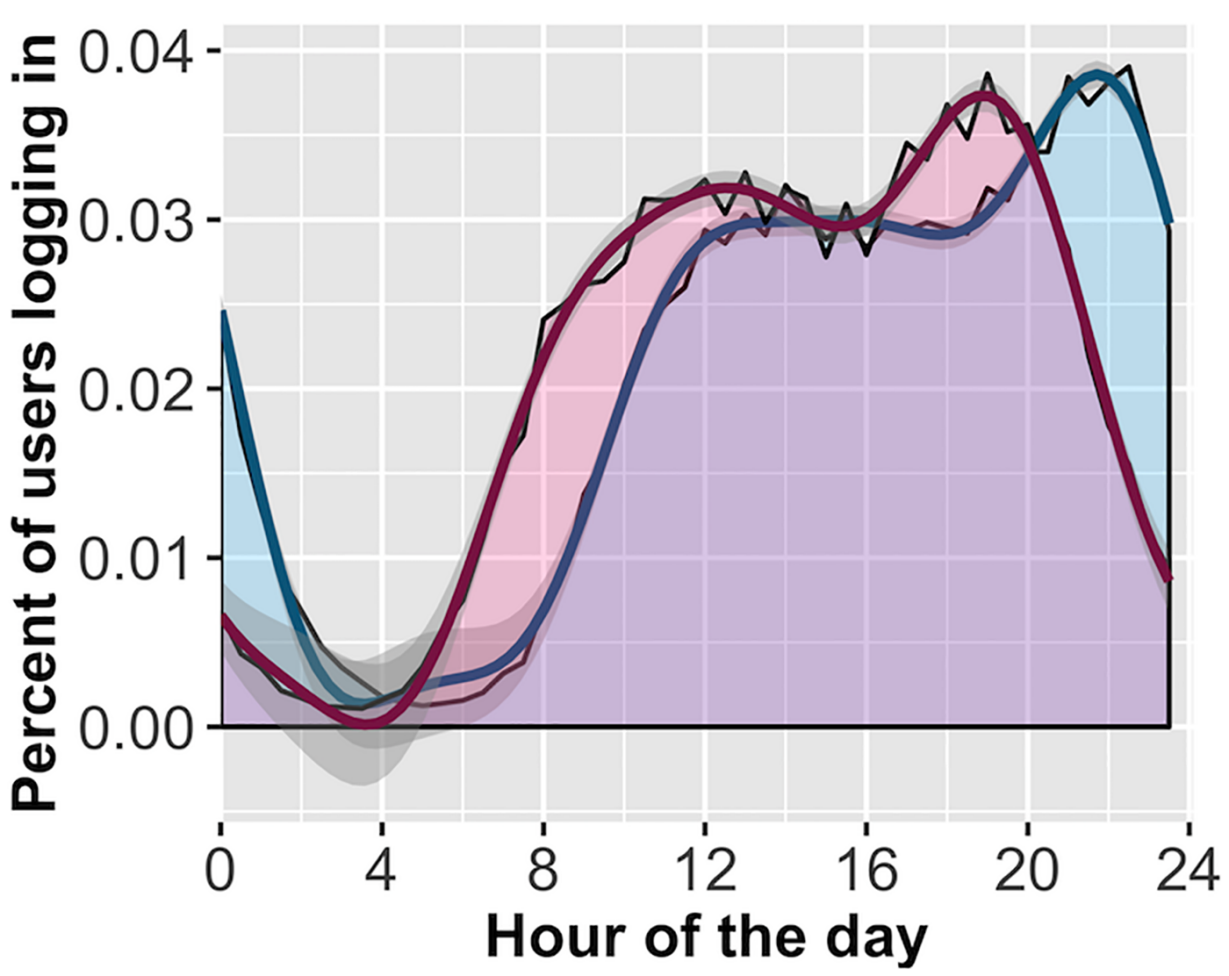
Login activity with 4th harmonic regression line. Notes: red is sample 1, blue is sample 2. Area plots displayed with data-fit 4th harmonic regression lines. Both samples exhibit a characteristic increase in login activity early in the day, followed by consistent activity that peaks late in the night. Sample 2 is adjusted for local time zone, which reveals login activity does not decline until around 2am. Black lines follow the density for each 30-minute window, colored lines represent regression function, grey shaded ribbons are 95% CI of the regression line.

In sample 1, we found that a 3rd harmonic sine function fit the data best (WAIC = -429.6), followed by the 4^th^ harmonic function (WAIC = -426.9). We ran an exact replication by using the pre-registered model specified by sample 1 to assess out-of-sample deviance in sample 2 (comparing RMSE). We again found that the 3rd harmonic sine function was the best fit (RMSE = 6.69 10^-6), though the 4th harmonic function fit nearly as well (RMSE = 6.73*10^-6). These models reduced out-of-sample error by almost 90% compared to the intercept-only model (intercept model RMSE = 6.51*10^-5). Finally, when re-running the models on sample 2 after adjusting for time-zones, we found that the 4th harmonic function (WAIC = -431.9) fit considerably better than other models, including the 3rd harmonic sine function (WAIC = -416.6; See S4 Table for full 4th harmonic regression model). The results strongly indicate that a multi-harmonic sine function is the best fit. Fig 6 indicates that login activity quickly increases in the morning and is consistent until later at night, when there is an additional spike in activity before dropping off almost completely at night.

Next, we fit our data to the three theoretical patterns of daily user behavior (Fig 2). We found that the model expecting an evening preference fit the data best. As a direct replication, we found our sample 2 data was also best described by this model, both without and with correcting for time-zone information (Table 2). The data from both samples strongly speak against a theoretical model that assumes people have a central reservoir of self-control from which to draw throughout the day.

**Table 2:** Deviance of login data from theoretical models of daily fluctuations in login patterns.

Model	Sample 1	Sample 2 direct replication	Sample 2 adjusting time zone
Limited SC	400.7	555.0	809.7
Unlimited SC	26.2	153.9	1715.1
Evening preference	-145.3	-217.0	178.6

Notes: SC = self-control. Values are the negative log likelihood of the data given the model. Smaller values imply less deviance and greater likelihood. See Fig 2 for representations of the theoretical models.

### Length of session based on time of day

**Fig 7:**
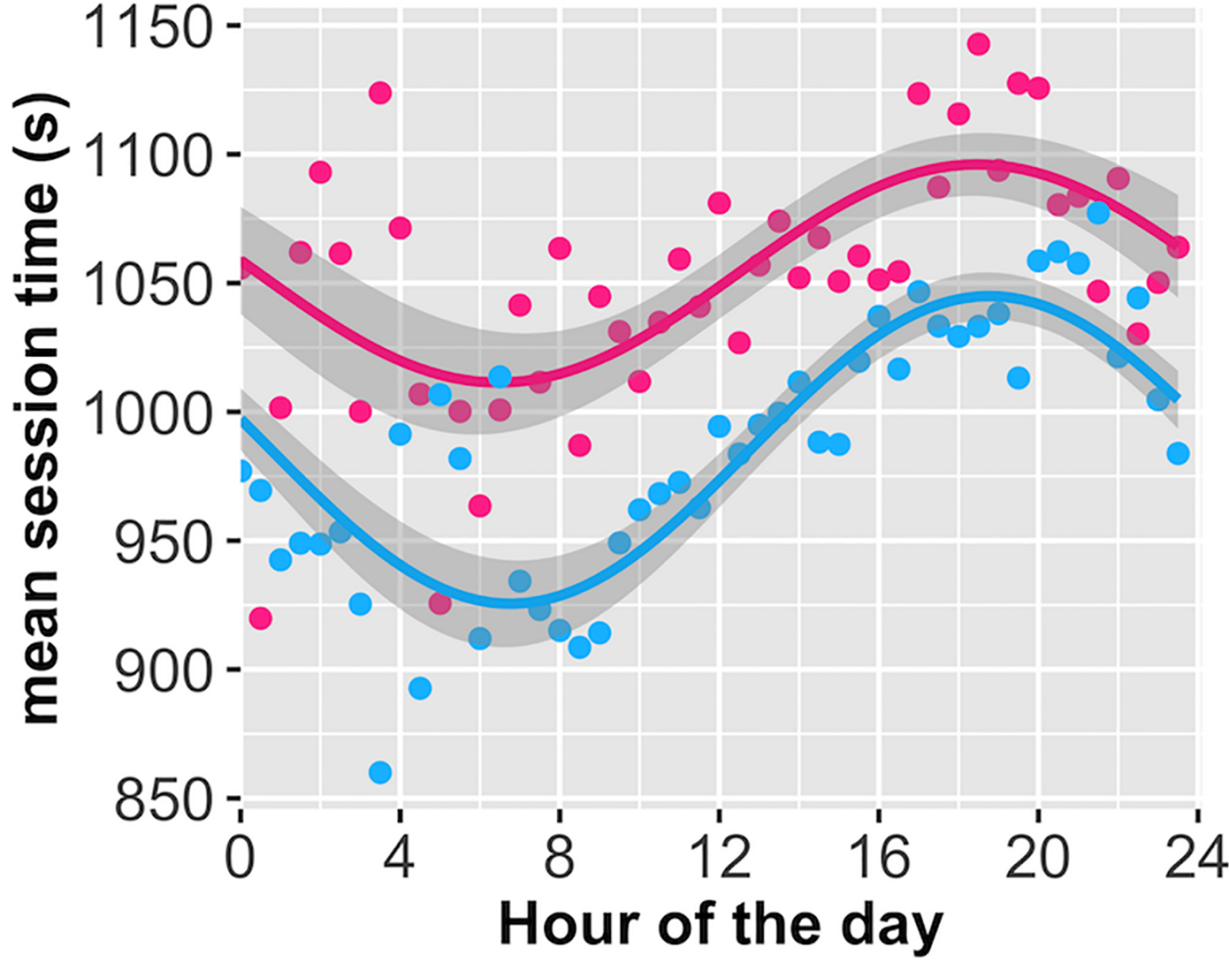
Average session length with 1st harmonic regression line. Red = sample 1 data. Blue = sample 2 adjusting for users’ time-zone. Points reflect the average session length across sessions for each 30-minute window. Colored lines represent regression line, shaded regions represent the 95% CI of the fixed effect.

As a different measure of motivation, we also looked at relative length of session instead of merely whether the user logged on. Cerego sessions are variable length based on how many “memories” a user still needs to learn or review. However, users are free to end their session early and leave remaining trials for a future session. Thus, if they are less motivated at certain times of day, their session length should be reduced. We employed the same analysis strategy as the previous section, with the exception that session length per time window was taken as the average length for all sessions beginning in that window. Again, comparing different models using WAIC, our initial analysis of sample 1 indicated that the 1st harmonic sine function was the best fit (WAIC = 491.9) with a score at least 10 points lower relative to other models. Out-of-sample deviance using sample suggested that the complexity of the sine wave is somewhat irrelevant for model fit, with the 2^nd^ harmonic fitting the best (RMSE = 3.91*10^3) and reducing out-of-sample by 21% relative to the intercept model (RMSE = 4.94*10^3). However, re-fitting the model again using sample 2 data after adjusting for time-zone indicates that a 1st harmonic sine wave is most parsimonious (WAIC = 467.8) with the lowest WAIC score by at least 11 points. As with login activity, we are confident that a sine function best describes session length, though there is some uncertainty regarding exactly which function fits best (Fig 7; S5 Table for full 1^st^ harmonic regression model).

Again, formally testing our data against the three theoretical models outlined in Fig 2, we find that sample 2 replicates our initial finding from sample 1; namely that the model assuming evening preference is most likely given the data (Table 3). As with login activity, the results suggest that people are motivated to complete their session at any time, but possibly have more time later in the evening to complete voluntary learning. The results do not support a theory of self-control where users’ central reservoir depletes throughout the day.

**Table 3:** Deviance of session length data from theoretical models of daily session length fluctuations.

Model	Sample 1	Sample 2 direct replication	Sample 2 adjusting time zone
Limited SC	2.8*10^3	2.0*10^3	2.4*10^3
Unlimited SC	3.1*10^3	2.0*10^3	3.0*10^3
Evening preference	1.2^10^3	8.0*10^2	9.3*10^2

Notes: Values are the negative log likelihood of the data given the model. Smaller values imply Notes: SC = self-control. Values are the negative log likelihood of the data given the model. Smaller values imply less deviance and greater likelihood. See Fig 2 for representations of the theoretical models.

## Discussion

We monitored two samples of students using the Cerego learning system over seventeen-week periods, to simultaneously assess within-task mental fatigue and domain-general self-control depletion in a naturalistic setting. From the theoretical position of resource models of self-control, we would expect that on average our sample would show increasing depletion throughout the day, and that this should translate into less motivation to complete learning sessions, as well as possibly worse performance. We found robust evidence that users decline in ability while persisting within task, but also strong evidence that domain-general depletion does not moderate this effect. While there was evidence that user ability did decline throughout the day, the magnitude of the effect was very small and appeared to be mostly related to the beginning of the session. Additionally, we find that user login activity and length of sessions strongly increases throughout the day and into the evening, with an abrupt decrease around times that people typically sleep. Overall the evidence is counter to predictions made by resource models of self-control.

Regarding within-task performance, users persisting through a session began to show a meaningful drop in performance by around 50 minutes OR 150 trials, similar to what is often found with mental fatigue studies [12]. This should be taken as a somewhat conservative estimate of the onset of declining ability, as we know that Cerego trials are presented in a non-random order, with more difficult trials displayed at the beginning of the session. This non-randomness may explain the apparent increase in ability after the first few trials and leaves open the possibility that the lowered performance could emerge in shorter time frames than we present. However, our results are fairly consistent with other naturalistic observations of within-domain fatigue in terms of both the time course and effect size (see Introduction).

Willingness to log in and length of session throughout the day speak against any effect of a depleting general resource. Users’ login patterns were steady throughout the day and increased substantially towards the evening, while their session lengths were longest later in the day relative to earlier. This is the exact opposite of what we would have expected from our predictions derived from the resource model of self-control, where a mentally effortful task should be unappealing later in the day. There are a few plausible interpretations of our results. One possibility is that time of day is relatively un-important for user motivation to complete a session, but that daytime responsibilities constrain them to work more at night. Another possibility, is that internal diurnal rhythms actually increase motivation for mentally stimulating tasks later in the day for young adults. Circadian rhythms have been shown to peak later in the afternoon for many young adults and are known to affect cognitive tasks that require control and attention [13–17]. Thus, our observed late-night peak in activity may be explained by increased alertness and preference for studying at this time. Even though we found very little variability in average score across time of day, our users may feel more motivated or believe that they are more effective studying later in the afternoon and evening. If this interpretation were correct, it would suggest that motivation is guided by internal rhythmic processes, and that external stressors requiring self-control were not relevant; i.e. the theorized causal process for depletion.

Despite showing motivation to complete learning sessions at night, participants did not show attendant increases in performance. Rather, they showed a small decrease in ability beginning in the early afternoon. Users show a steady decline in ability beginning at around 2pm once data are adjusted for local time zones. However, two issues limit our ability to interpret this as evidence for ego depletion. For one, the drop in performance is trivial compared to what we see with within-session mental fatigue. If our effect is being generated by the same process that leads to the large ego depletion effect often seen in experiments over minutes [4], there is considerable work needed to explain why the effect is large in such brief time scales, and so small at longer ones. An additional concern is the time course. We would have anticipated a decrease in performance beginning as early as 10am, as with the Danish student study [9] and in line with our simulations. An early afternoon peak of session score, followed by a decline into the evening, possibly again points to homeostatic and circadian cycles as the causal mechanism, as opposed to daily stressors. Finally, when looking at the individual parameters of the within-session model, the one parameter most sensitive to time of day was the intercept, speaking to the users’ ability to answer one-shot relatively difficult questions at the beginning of their session. We would have expected to see a moderation of the linear or quadratic components of this model if time of day was influencing depletion, but no significant effect was present and the effects were inconsistent across the two samples. Further, this moderation was quite small.

One limitation of our analysis, is that we did not control for user-level behavior in our time-of-day analyses. Thus, there is a potential confound, where users who prefer later sessions may also have been likely to spend more time or perform slightly worse. We pre-registered our approach based on concerns that many users provided an insufficient number of sessions, or sessions clustered too tightly around certain time periods. Given we were testing for multi-harmonic sine functions, the data would have been inappropriate. An additional important limitation of our work, is that we do not have information regarding who our sample are, what individual differences might be present, and how they viewed the activity. These limitations were a necessity to access the information, but it prevents us from making strong positive interpretations of the pattern of results. However, while additional work would be needed to offer a complete explanation of our findings, we believe that it is very difficult to interpret these findings as supportive of the resource model of self-control, and in fact offer compelling counter-evidence of the existence of domain-general depletion. First, we have strong evidence for within-task declines in performance that emerge after about 50 minutes of continuous performance, consistent with other large and detailed datasets. Second, we have only weak or inconsistent evidence that time-of-day impacts performance; the notion that this fatigue is completely fluid, and that it emerges after minutes of self-control, is under considerable doubt.

## Supporting information

S1 Text Simulation of average self-control depletion for a population throughout the day. (DOCX)

S2 Text Data screening and preparation. (DOCX)

S3 Text Defining theoretical models of self-control. (DOCX)

S1 Table Accuracy as a function of trial number for samples 1 and 2. (DOCX)

S2 Table Accuracy as a function of elapsed time for samples 1 and 2. (DOCX)

S3 Table 1^st^ harmonic regression model of session accuracy for samples 1 and 2. (DOCX)

S4 Table 4^th^ harmonic regression model of login time for samples 1 and 2. (DOCX)

S5 Table 1^st^ harmonic regression model of session length for samples 1 and 2. (DOCX)

S6 Table Regressing the session start coefficient (intercept term) onto time-of-day. (DOCX)

S7 Table Regressing the linear effect coefficient of session length onto time-of-day. (DOCX)

S8 Table Regressing the quadratic effect coefficient of session length on time-of-day. (DOCX)

S9 Table Accuracy as a function of trial for samples 1 and 2 –Nested within session within user. (DOCX)
