# Supplementary material for: A pre-registered naturalistic observation of within domain mental fatigue and domain-general depletion of self-control
Source: PLoS One. 2017 Sep 20;12(9):e0182980. doi: 10.1371/journal.pone.0182980 (PMC5607124; doi:10.1371/journal.pone.0182980)
Supplement: S1 Text — (DOCX) [file pone.0182980.s001.docx]

**S1 Text**

**Simulation of average self-control depletion for a population throughout the day**

The figures below demonstrate average self-control reserves in a population, under the assumption that people experience domain-general self-control depletion when they need to actively control themselves. The simulations that generate these figures make the following assumptions:

- Everyone wakes at 9am with full capacity to engage self-control.
- Events requiring self-control occur every half-hour with some chance. These events last between 30-minutes to 3 hours, with every 30-minute increment depleting self-control by 10% the individual’s current reserves. The implication is that as people become depleted, they use less self-control in situations that demand it (presumably worsening their performance in the moment).
- If an individual is already engaged in an event requiring self-control, a new event will not start. The assumption is that we can only engage in one task at a time.
- If an individual is not using self-control and no event begins in a particular window, there is a chance they will have the opportunity to engage in a rest event. Rest events rejuvenate self-control by a full 10% (regardless of their current levels) but not more than 100%. This assumes that rest-events are always maximally rejuvenating. In combination with reduced use of self-control when an individual has low reserves, this creates a scenario where individuals can function throughout the day without “crashing”. For example, if an individual is at 50%, a self-control event would only lower their reserves by 5%, but a rest-event will replenish it by 10%.

In the first simulation (Figure S.1.1 a), we assume that most people have a high chance of not using self-control, and many opportunities to engage in restful activities. Despite individuals only experiencing on average 5.45 hours of events that require self-control (SD = .29 across simulations) and spending 7.3 hours a day replenishing (SD = .04 hours), the average levels of self-control still drop throughout the day, particularly in the first few hours after waking. Arguably, most young adults spend at least more time engaging in tasks that require self-control than they do on rest during any given day. In panel b, we assume that most people have a higher chance of using self-control, with somewhat fewer opportunities to engage in restful activities. People spend an average of 7.37 hours using self-control (SD = .32 hours across simulations) and 4.35 hours resting (SD = .04 hours). We would suggest that this scenario more plausibly reflects a young-adult’s average day. In this scenario, the average self-control an individual has access to drops continuously until the afternoon.

**Figure – Simulation output**

1. *b)*

**
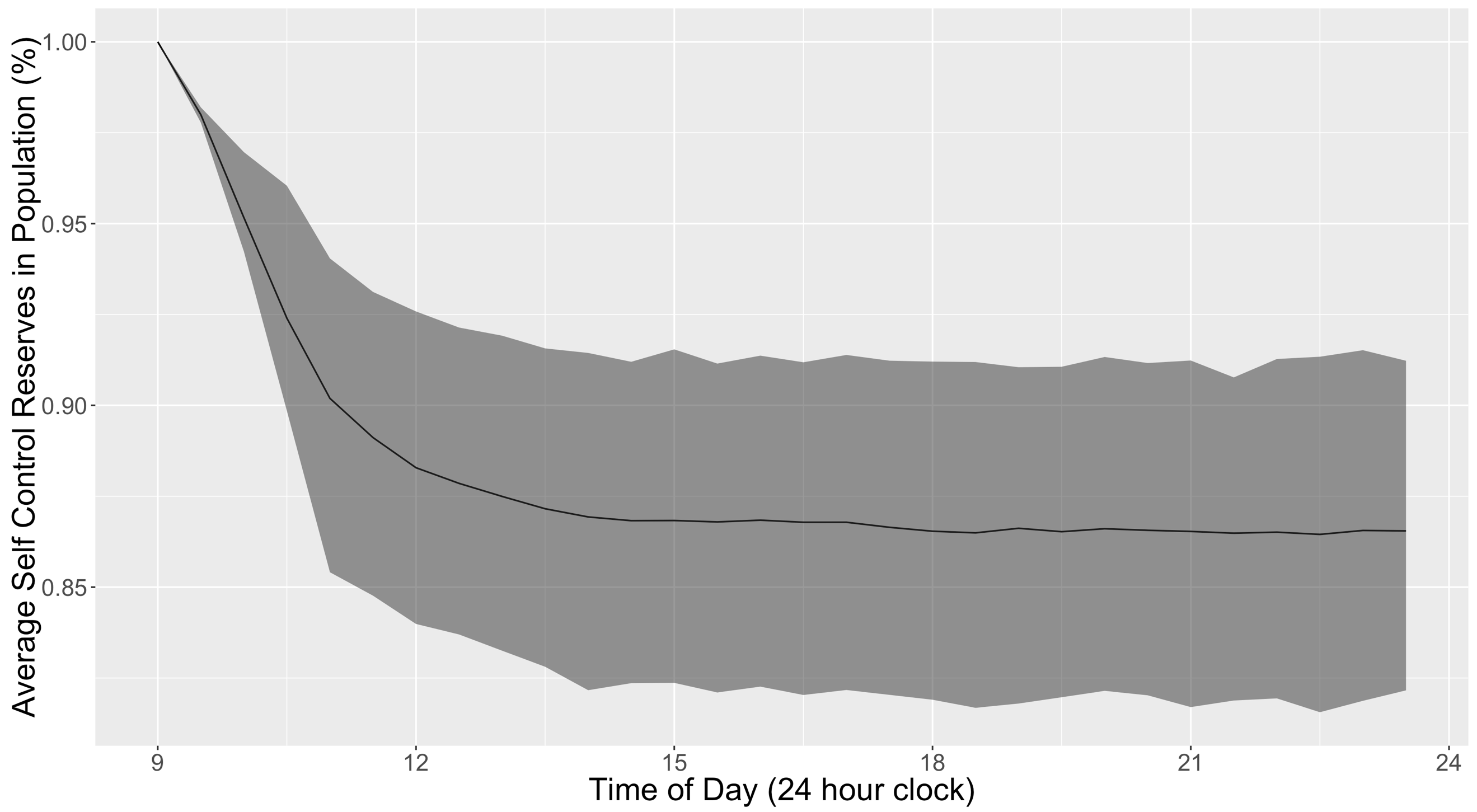
**
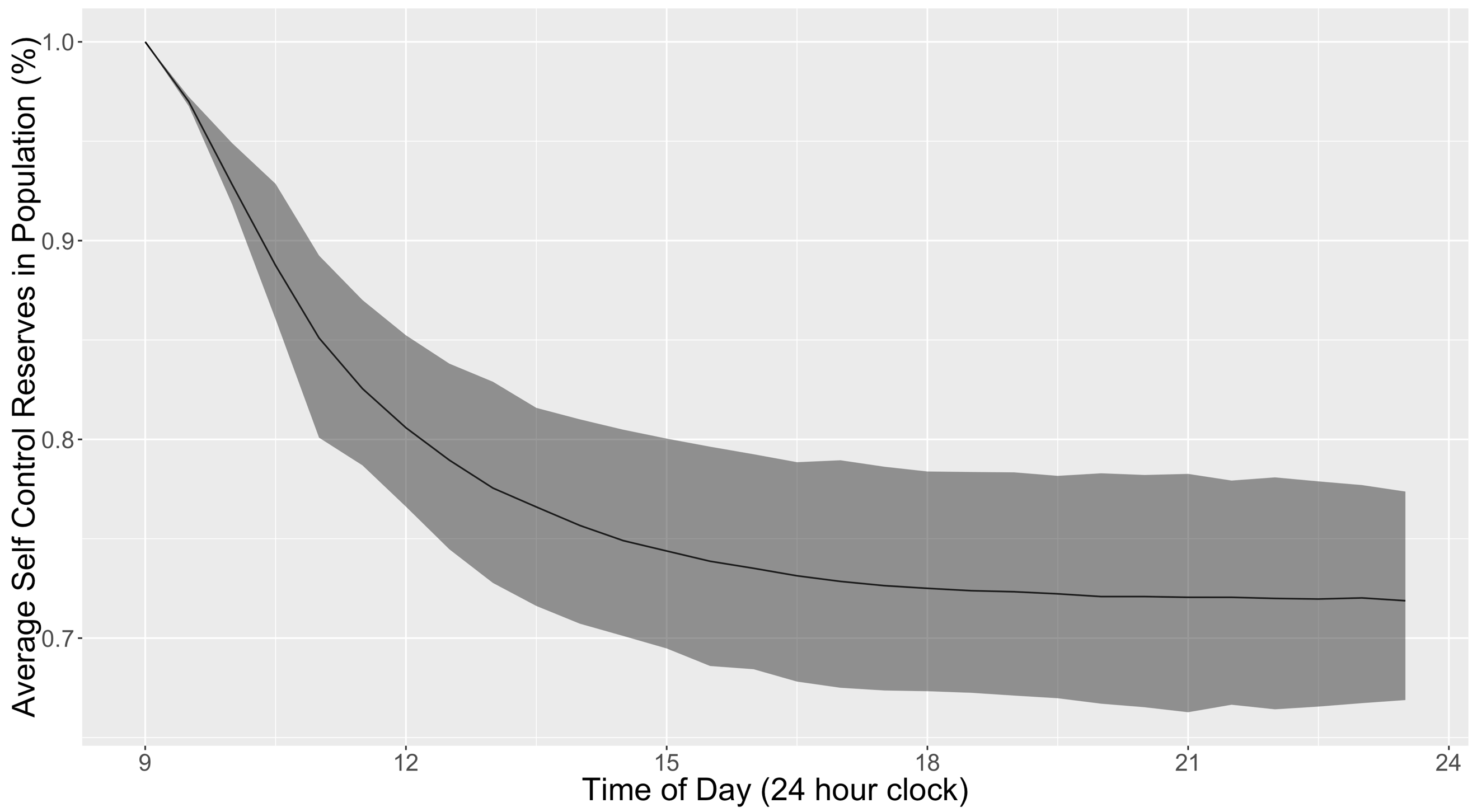


Output from the simulation for a 24-hour period. a) A simulation where individuals have mostly relaxed days. 20% chance of events requiring self-control in any 30-minute window. 50% chance of rest opportunity if no event required self-control. b) A simulation where individuals have more active days. 30% chance of an event requiring self-control, 30% chance of an opportunity to rest if no self-control was required. Black line represents the average of a 1000 simulations, shaded region represents values for 95% of all simulations.

Simulation

# Shows how a population's self-control would deplete on average throughout # the day, under different assumptions of work/rest time. Black line # represents the average of a 1000 simulations, each with a population of # 1000 people. Shaded region represents the range that 95% of all simulations # fall within.

# Packages needed for the script to run properly.
library(tidyr)
library(plyr)
library(dplyr)
library(ggplot2)
library(lazyeval)

# Variables that can be adjusted to change the simulation constraints

depletion <- c(rep('Y', 300), rep('N', 700))

# % chance of a stressful event at any given 30-minute window. Default here # is 30%. Y represents a stressful event N represents no event. Both values # should sum to 1000.

length_of_depleting_event <- seq(0,3)

# How long can stressful events last. Default is 1 - 4 30-minute increments. # Increase the lower bound by changing 0 to a larger number. Increase or # decrease the upper bound by changing 3.

rest <- c(rep('Y', 300), rep('N', 700))

# % chance of a rest event at any given time 30-minute window. Default here # is 30%. Y represents a rest event N represents no event. Both values should # sum to 1000.

# Code below this line does not need to be adjusted. Use the variables above # to adjust simulation parameters. Run the entire script to generate results.
###############################################################

# Priming variables for multiple simulations
sims_times <- paste(rep('time', 30), seq(1,30), sep = '')
meta_output <- as.data.frame(setNames(replicate(30,numeric(0)), sims_times))
meta_event_mean <- c()
meta_rest_mean <- c()

for (j in 1:1000){ # 1000 simulations are run.

# Priming variables for an individual simulation
time1 <- rep(1, 1000)
sims <- as.data.frame(time1)
for (i in 2:30) {
 sims[[sims_times[i]]] <- rep(1, 1000)
}
event_counter <- rep(0, 1000)
event_time_names <- paste(rep('event_time'), seq(1, 30), sep = '')

# Simulation of a single day. Every half hour There is a chance of stressful # event beginning, a stressful event continuing, a rest event beginning, or # no event.
for (i in 1:29){
 depletion_temp <- sample(depletion, 1000, replace = TRUE)
 sims[[event_time_names[i]]] <- ifelse(event_counter > 0,

'Y', depletion_temp)

# If stressful event begins or continues, assign Y.
 event_counter <- ifelse(event_counter == 0 & depletion_temp == 'Y',

sample(length_of_depleting_event, size = 1),

# Draw how long the event lasts.
 ifelse(event_counter > 0, event_counter - 1, 0)) # Reduce length of on-going stressful event from previous window.
}

rest_time_names <- paste(rep('rest_time'), seq(1,30), sep = '')
for (i in 1:29){
 sims[[rest_time_names[i]]] <- sample(rest, 1000, replace = TRUE)
}

# Generate data frame of self-control reserves for the simulated population.
for (i in 2:30){
 sims[[sims_times[i]]] <- ifelse(

sims[[event_time_names[i- 1]]] == 'Y', sims[[sims_times[i - 1]]]*.9,
# If stressful event occurred in previous window, current self-control by 10%
 ifelse(sims[[sims_times[i - 1]]] >= 1, sims[[sims_times[i - 1]]], # If no stressful event and reserves are full, no change.
 ifelse(sims[[rest_time_names[i - 1]]] == 'Y' & sims_times[i - 1] < .9,

sims[[sims_times[i - 1]]] + .1,
# If no stressful event and reserves are less than .9, increase by .1
 ifelse(sims[[rest_time_names[i - 1]]] == 'Y' & sims_times[i - 1] >= .9,

1, sims[[sims_times[i - 1]]]))))
# If greater than .9, return reserves to full i.e. 1.
}

# Summary variables from a single simulation
sims$total_self_control_events <- rep(0, 1000)
sims$total_rest_events <- rep(0,1000)
event_time_names_2 <- paste('sims$', event_time_names, sep ='')
rest_time_names_2 <- paste('sims$', rest_time_names, sep = '')
x <- c()
for (i in 1:29){
 sims$total_self_control_events <- ifelse(

eval(parse(text = event_time_names_2[i])) == 'Y',

sims$total_self_control_events + 1, sims$total_self_control_events)
 sims$total_rest_events <- ifelse(

eval(parse(text = rest_time_names_2[i])) == 'Y',

sims$total_rest_events + 1, sims$total_rest_events)
}
rm(event_time_names_2, rest_time_names_2)
single_sim_output <- sims %>% select(starts_with('time')) %>% summarise_each(funs(mean))
single_sim_event_mean <- mean(sims$total_self_control_events)
single_sim_rest_mean <- mean(sims$total_rest_events)

# Add summary values from single simulation to the meta-counters.
meta_output <- rbind(meta_output, single_sim_output)
meta_event_mean <- c(meta_event_mean, single_sim_event_mean)
meta_rest_mean <- c(meta_rest_mean, single_sim_rest_mean)
} # End of loop running 1000 versions of the simulation.

# End of outer loop, summarizing values from the 1000 simulations

lower_quantile <- function(x = NULL){
 output <- quantile(x = x, prob = .05, names = FALSE)
 return(output)
}

upper_quantile <- function(x = NULL){
 output <- quantile(x = x, prob = .95, names = FALSE)
 return(output)
}

summary_data <- meta_output %>%

summarise_each(funs(mean, lower_quantile, upper_quantile))

# Organizes summary data into a data frame.
mean_list <- c()
lower_quantile_list <- c()
upper_quantile_list <- c()
for (i in 1:30){
 mean_list <- c(mean_list, summary_data[[i]])
 lower_quantile_list <- c(lower_quantile_list, summary_data[[i+30]])
 upper_quantile_list <- c(upper_quantile_list, summary_data[[i+60]])
}

sim_output <- data.frame(

mean = mean_list,

fifth_quantile = lower_quantile_list,

ninety_fifth_quantile = upper_quantile_list)


# Plotting the figure
time_list <- seq(from = 9, to = 23.5, by = .5)
sim_output <- cbind(sim_output, time_list)
ggplot(sim_output, aes(x = time_list, y=mean)) +
 geom_line() +
 geom_ribbon(aes(ymin = fifth_quantile, ymax = ninety_fifth_quantile,

colour = 'grey', alpha = .2), colour = NA) +
 ylab('Average Self Control Reserves in Population (%)') +

xlab('Time of Day (24 hour clock)') +
 scale_x_continuous(breaks = c(9, 12, 15, 18, 21, 24),

labels = c(9, 12, 15, 18, 21, 24)) +
 theme(legend.position = 'none', axis.title = element_text(size = 20),

axis.text = element_text(size = 15))
