# Supplementary material for: A pre-registered naturalistic observation of within domain mental fatigue and domain-general depletion of self-control
Source: PLoS One. 2017 Sep 20;12(9):e0182980. doi: 10.1371/journal.pone.0182980 (PMC5607124; doi:10.1371/journal.pone.0182980)
Supplement: S2 Text — (DOCX) [file pone.0182980.s002.docx]

**S2 Text**

**Data screening and preparation**

Data screening procedures were set based on our understanding of the Cerego system and inspection of various outliers in distributions. Screening procedures were exploratory in sample 1, but fixed for sample 2 (see pre-registration, http://tinyurl.com/zby5pzt). The datasets contained many users who created accounts with Cerego but did not continue use after trying it out. These users have logged sessions, but were likely exploring the system and not providing useful data for our purposes. Only participants who had completed at least 5 sessions were included in the analyses, which removed roughly 8% of the initial sample in sample 1 and 5% in sample 2.

Additionally, we wanted to use as many sessions as possible, while also eliminating sessions where an individual had walked away from the program and returned after a break. To address this, we removed sessions that lasted for more than 3600 seconds (one hour), which eliminated 10% of the remaining sample 1 and 11% for sample 2. We also divided each session time by the number of trials to estimate average time-per-trial within session, with the aim of removing participants who either moved too quickly or lingered long enough to suggest they weren’t actively using the program. Quantile analysis indicated most participants spent 11 to 24 seconds (1^st^ and 3^rd^ quartile) per trial. A small percentage of sessions involved less than 1 second per trial (roughly 1% of the sample) and roughly 1% of the sessions had very long time-per-trials, averaging 426 seconds per trial. Sessions belonging to either of these groups were removed.

The analyses of trial order on accuracy had additional restrictions. To minimize the effects of outliers and qualitatively different sessions, we removed any trials that went beyond 225, and entire sessions that lasted fewer than 50 trials.

After these exclusions, the final dataset for sample 1 contained 8867 users completing 72082 sessions and sample 2 contained 8754 users completing 147186 sessions. For sample 1, each user completed an average of 13.36 sessions, with each session lasting for an average of 65.82 trials and 1196 seconds (just under 20 minutes). For sample 2, each user completed an average of 16.81 sessions, with each session lasting an average of 58.8 trials and 1006 seconds.
