# Supplementary material for: A pre-registered naturalistic observation of within domain mental fatigue and domain-general depletion of self-control
Source: PLoS One. 2017 Sep 20;12(9):e0182980. doi: 10.1371/journal.pone.0182980 (PMC5607124; doi:10.1371/journal.pone.0182980)
Supplement: S3 Text — (DOCX) [file pone.0182980.s003.docx]

**S3 Text**

**Defining theoretical models of self-control**

For each analysis, we compared how well each of three theoretical models of behavior could account for our data. We wanted to maintain the conceptual structure of the model for each analysis, while adjusting the parameters to be sensible for each particular analysis. In general, all three models were constructed with the following criteria and were pre-registered for sample 2: minimum activity is from 10pm - 6am, defined as the average value of all time windows in this period; peak activity is based on the highest average value for any 30-minute window, standard deviation of the model is equal to 10% of the range between the minimum and the peak. For each specific analysis, parameters were set as follows:

*Login time*: For sample 1, minimum value = .0056, max = .0386, SD = .0033; for sample 2 without adjusting for time-zone data, minimum = .0071, max = .0380, SD = .0031; for sample 2 after adjusting for time-zone data, minimum = .0143, max = .0391, SD = .0025.

*Session Length*: For sample 1, minimum value = 1035 seconds, max = 1143, SD = 10.8; for sample 2 without adjusting for time-zone data, minimum = 945, max = 1064, SD = 11.9; for sample 2 after adjusting for time-zone data, minimum = 966, max = 1077, SD = 11.1.

*Aacuracy*: For sample 1, minimum value = 86%, max = 88%, SD = .2; For sample 2 without adjusting time-zone, minimum = 86%, max = 88%, SD = .2; For sample 2 after adjusting time-zone, minimum = 86%, max = 88%, SD = .2.
