# Supplementary material for: A pre-registered naturalistic observation of within domain mental fatigue and domain-general depletion of self-control
Source: PLoS One. 2017 Sep 20;12(9):e0182980. doi: 10.1371/journal.pone.0182980 (PMC5607124; doi:10.1371/journal.pone.0182980)
Supplement: S1 Table — (DOCX) [file pone.0182980.s004.docx]

**S1 Table**

**Accuracy as a function of trial number for samples 1 and 2**

|  |  | Sample 1 accuracy | | |  | Sample 2 accuracy | | |
| --- | --- | --- | --- | --- | --- | --- | --- | --- |
|  |  | *B* | *CI* | *p* |  | *B* | *CI* | *p* |
| **Fixed Parts** | | | | | | | | |
| (Intercept) |  | 0.8085 | 0.8053 – 0.8116 | **<.001** |  | 0.8154 | 0.8128 – 0.8180 | **<.001** |
| Trials (linear) |  | 0.0776 | 0.0717 – 0.0835 | **<.001** |  | 0.0800 | 0.0756 – 0.0844 | **<.001** |
| Trials (quadratic) |  | -0.0549 | -0.0588 – -0.0509 | **<.001** |  | -0.0584 | -0.0615 – -0.0553 | **<.001** |
| **Random Parts** | | | | | | | | |
| σ^2^ |  | 0.051 | | |  | 0.048 | | |
| τ_00, user_ |  | 0.012 | | |  | 0.012 | | |
| ρ_01_ |  | -0.353 | | |  | -0.311 | | |
| N_user_ |  | 5566 | | |  | 8544 | | |
| ICC_user_ |  | 0.189 | | |  | 0.204 | | |
| Observations |  | 738946 | | |  | 1354597 | | |
| R^2^ / Ω_0_^2^ |  | .239 / .239 | | |  | .239 / .238 | | |

Notes: MLM with random slope and intercept within user. Dependent variable is the average correct score within a cluster of five trials. Independent variable is the order of trial clusters.
