# Supplementary material for: A pre-registered naturalistic observation of within domain mental fatigue and domain-general depletion of self-control
Source: PLoS One. 2017 Sep 20;12(9):e0182980. doi: 10.1371/journal.pone.0182980 (PMC5607124; doi:10.1371/journal.pone.0182980)
Supplement: S2 Table — (DOCX) [file pone.0182980.s005.docx]

**S2 Table**

**Accuracy as a function of elapsed time for samples 1 and 2**

|  |  | Sample 1 accuracy | | |  | Sample 2 accuracy | | |
| --- | --- | --- | --- | --- | --- | --- | --- | --- |
|  |  | *B* | *CI* | *p* |  | *B* | *CI* | *p* |
| **Fixed Parts** | | | | | | | | |
| (Intercept) |  | 0.8016 | 0.7981 – 0.8050 | **<.001** |  | 0.8086 | 0.8058 – 0.8114 | **<.001** |
| Elapsed time (linear) |  | 0.5433 | 0.5037 – 0.5829 | **<.001** |  | 0.5140 | 0.4860 – 0.5420 | **<.001** |
| Elapsed time (quadratic) |  | -1.7316 | -1.8824 – -1.5807 | **<.001** |  | -1.5511 | -1.6562 – -1.4460 | **<.001** |
| **Random Parts** | | | | | | | | |
| σ^2^ |  | 0.051 | | |  | 0.049 | | |
| τ_00, user_ |  | 0.015 | | |  | 0.015 | | |
| ρ_01_ |  | -0.524 | | |  | -0.470 | | |
| N_user_ |  | 5566 | | |  | 8544 | | |
| ICC_user_ |  | 0.228 | | |  | 0.240 | | |
| Observations |  | 738946 | | |  | 1397767 | | |
| R^2^ / Ω_0_^2^ |  | .240 / .240 | | |  | .244 / .243 | | |

Notes: MLM with random slope and intercept within user. Dependent variable is the average correct score within a cluster of five trials. Independent variable is the average elapsed time per trial cluster. This analysis is similar to Table S2 but accounts for variation of time spent on trials.
