# Supplementary material for: A pre-registered naturalistic observation of within domain mental fatigue and domain-general depletion of self-control
Source: PLoS One. 2017 Sep 20;12(9):e0182980. doi: 10.1371/journal.pone.0182980 (PMC5607124; doi:10.1371/journal.pone.0182980)
Supplement: S3 Table — (DOCX) [file pone.0182980.s006.docx]

**S3 Table**

**1st harmonic regression model of session accuracy for sample 1 and 2**

|  |  | Sample 1 accuracy | | | |  | Sample 2 accuracy | | | |
| --- | --- | --- | --- | --- | --- | --- | --- | --- | --- | --- |
|  |  | *B* | *CI* | *SE* | *p* |  | *B* | *CI* | *SE* | *p* |
| (Intercept) |  | 0.8525 | 0.8509 – 0.8540 | 0.00 | **<.001** |  | 0.8658 | 0.8645 – 0.8671 | 0.00 | **<.001** |
| Sine 1 |  | 0.0010 | -0.0010 – 0.0029 | 0.00 | .320 |  | -0.0014 | -0.0033 – 0.0005 | 0.00 | .149 |
| Cos 1 |  | -0.0030 | -0.0050 – -0.0010 | 0.00 | **.005** |  | -0.0047 | -0.0060 – -0.0033 | 0.00 | **<.001** |
| time windows |  | 48 | | | |  | 48 | | | |

Notes: The time-zones of users for sample 1 are censored. Data for sample 2 have been adjusted for user time-zones. Individual regression components are not meaningful, but both composite regression lines are presented in Figure 4 in the main document. Dependent variable is the average session accuracy for any session starting in half-hour windows, with an intercept set at midnight.
