# Supplementary material for: A pre-registered naturalistic observation of within domain mental fatigue and domain-general depletion of self-control
Source: PLoS One. 2017 Sep 20;12(9):e0182980. doi: 10.1371/journal.pone.0182980 (PMC5607124; doi:10.1371/journal.pone.0182980)
Supplement: S4 Table — (DOCX) [file pone.0182980.s007.docx]

**S4 Table**

**4th harmonic regression model of login time for samples 1 and 2.**

|  |  | Sample 1 login time | | | |  | Sample 2 login time | | | |
| --- | --- | --- | --- | --- | --- | --- | --- | --- | --- | --- |
|  |  | *B* | *CI* | *SE* | *p* |  | *B* | *CI* | *SE* | *p* |
| (Intercept) |  | 0.0208 | 0.0201 – 0.0215 | 0.00 | **<.001** |  | 0.0209 | 0.0203 – 0.0215 | 0.00 | **<.001** |
| Sine 1 |  | -0.0122 | -0.0132 – -0.0113 | 0.00 | **<.001** |  | -0.0159 | -0.0169 – -0.0149 | 0.00 | **<.001** |
| Cos 1 |  | -0.0112 | -0.0122 – -0.0102 | 0.00 | **<.001** |  | -0.0016 | -0.0022 – -0.0010 | 0.00 | **<.001** |
| Sine 2 |  | -0.0059 | -0.0069 – -0.0049 | 0.00 | **<.001** |  | -0.0046 | -0.0053 – -0.0039 | 0.00 | **<.001** |
| Cos 2 |  | -0.0015 | -0.0023 – -0.0007 | 0.00 | **<.001** |  | 0.0053 | 0.0045 – 0.0062 | 0.00 | **<.001** |
| Sine 3 |  | 0.0015 | 0.0007 – 0.0023 | 0.00 | **.001** |  | -0.0028 | -0.0035 – -0.0021 | 0.00 | **<.001** |
| Cos 3 |  | -0.0013 | -0.0022 – -0.0005 | 0.00 | **.002** |  | -0.0004 | -0.0011 – 0.0003 | 0.00 | .216 |
| Sine 4 |  | 0.0016 | 0.0009 – 0.0023 | 0.00 | **<.001** |  | -0.0019 | -0.0025 – -0.0014 | 0.00 | **<.001** |
| Cos 4 |  | -0.0002 | -0.0009 – 0.0005 | 0.00 | .651 |  | 0.0005 | -0.0001 – 0.0010 | 0.00 | .124 |
| time windows |  | 48 | | | |  | 48 | | | |
| R^2^ / adj. R^2^ |  | .976 / .971 | | | |  | .983 / .979 | | | |

Notes: The time-zones of users for sample 1 are censored. Data for sample 2 have been adjusted for user time-zones. Individual regression components are not meaningful, but both composite regression lines are presented in Figure 6 in the main article. Dependent variable is the relative density of login times for any half-hour period, beginning at midnight.
