# Supplementary material for: A pre-registered naturalistic observation of within domain mental fatigue and domain-general depletion of self-control
Source: PLoS One. 2017 Sep 20;12(9):e0182980. doi: 10.1371/journal.pone.0182980 (PMC5607124; doi:10.1371/journal.pone.0182980)
Supplement: S5 Table — (DOCX) [file pone.0182980.s008.docx]

**S5 Table**

**1st harmonic regression model of session length for samples 1 and 2**

|  |  | Sample 1 average session length | | | |  | Sample 2 average session length | | | |
| --- | --- | --- | --- | --- | --- | --- | --- | --- | --- | --- |
|  |  | *B* | *CI* | *SE* | *p* |  | *B* | *CI* | *SE* | *p* |
| (Intercept) |  | 1053.70 | 1043.40 – 1064.01 | 5.12 | **<.001** |  | 985.24 | 977.61 – 992.87 | 3.79 | **<.001** |
| Sine 1 |  | -42.03 | -55.35 – -28.72 | 6.61 | **<.001** |  | -58.52 | -70.16 – -46.88 | 5.78 | **<.001** |
| Cos 1 |  | 5.10 | -8.62 – 18.83 | 6.82 | .458 |  | 11.90 | 3.62 – 20.17 | 4.11 | **.006** |
| time windows |  | 48 | | | |  | 48 | | | |
| R^2^ / adj. R^2^ |  | .529 / .508 | | | |  | .734 / .722 | | | |

Notes: The time-zones of users for sample 1 are censored. Data for sample 2 have been adjusted for user time-zones. Individual regression components are not meaningful, but both composite regression lines are presented in Figure 7 in the main document. Dependent variable is the average length of study session for any session starting in half-hour periods, with the intercept set at midnight.
