# Supplementary material for: A pre-registered naturalistic observation of within domain mental fatigue and domain-general depletion of self-control
Source: PLoS One. 2017 Sep 20;12(9):e0182980. doi: 10.1371/journal.pone.0182980 (PMC5607124; doi:10.1371/journal.pone.0182980)
Supplement: S6 Table — (DOCX) [file pone.0182980.s009.docx]

**S6 Table**

**Regressing the session start (intercept term) onto time-of-day**

|  |  | Sample 1 | | | |  | Sample 2 | | | |
| --- | --- | --- | --- | --- | --- | --- | --- | --- | --- | --- |
|  |  | *B* | *CI* | *SE* | *p* |  | *B* | *CI* | *SE* | *p* |
| (Intercept) |  | 0.8049 | 0.8036 – 0.8063 | 0.00 | **.0000** |  | 0.8140 | 0.8131 – 0.8150 | 0.00 | **.0000** |
| time-of-day(Sine 1) |  | 0.0010 | -0.0007 – 0.0028 | 0.00 | .2474 |  | 0.0010 | -0.0004 – 0.0025 | 0.00 | .1678 |
| time-of-day(Cos 1) |  | -0.0027 | -0.0045 – -0.0009 | 0.00 | **.0030** |  | -0.0040 | -0.0050 – -0.0030 | 0.00 | **.0000** |
| time windows |  | 39201 | | | |  | 72719 | | | |

Notes: The dependent variable is the intercept for each regression of trial number on trial accuracy. It reflects the average value of the first 1-5 trials for each session. A significant effect for time-of-day (in the cosine component) indicates an interaction, such that the intercept value for each session fluctuates as a function of time of day. The effect is consistent in both samples.
