# Supplementary material for: A pre-registered naturalistic observation of within domain mental fatigue and domain-general depletion of self-control
Source: PLoS One. 2017 Sep 20;12(9):e0182980. doi: 10.1371/journal.pone.0182980 (PMC5607124; doi:10.1371/journal.pone.0182980)
Supplement: S8 Table — (DOCX) [file pone.0182980.s011.docx]

**S8 Table**

**Regressing the quadratic effect coefficient of session length on time-of-day**

|  |  | Sample 1 | | | |  | Sample 2 | | | |
| --- | --- | --- | --- | --- | --- | --- | --- | --- | --- | --- |
|  |  | *B* | *CI* | *SE* | *p* |  | *B* | *CI* | *SE* | *p* |
| (Intercept) |  | -0.0486 | -0.0500 – -0.0472 | 0.00 | **.0000** |  | -0.0486 | -0.0497 – -0.0475 | 0.00 | **.0000** |
| time-of-day(Sine 1) |  | -0.0015 | -0.0033 – 0.0003 | 0.00 | .1121 |  | 0.0019 | 0.0002 – 0.0036 | 0.00 | **.0259** |
| time-of-day(Cos 1) |  | 0.0003 | -0.0015 – 0.0022 | 0.00 | .7156 |  | -0.0004 | -0.0016 – 0.0008 | 0.00 | .4995 |
| time windows |  | 39201 | | | |  | 72719 | | | |

Notes: The dependent variable is the quadratic beta coefficient for each regression of trial number on session. It reflects the quadratic effect of trial number on session accuracy. In this analysis, there is no significant effect at our adjusted threshold (p< .008) and the pattern of effects is not consistent between samples (see plot).
