# Supplementary material for: A pre-registered naturalistic observation of within domain mental fatigue and domain-general depletion of self-control
Source: PLoS One. 2017 Sep 20;12(9):e0182980. doi: 10.1371/journal.pone.0182980 (PMC5607124; doi:10.1371/journal.pone.0182980)
Supplement: S9 Table — (DOCX) [file pone.0182980.s012.docx]

**S9 Table**

|  |  | Sample 1 accuracy | | | |  | Sample 2 accuracy | | | | |
| --- | --- | --- | --- | --- | --- | --- | --- | --- | --- | --- | --- |
|  |  | *B* | *CI* | *SE* | *p* |  | *B* | *CI* | *SE* | *p* | |
| **Fixed Parts** | | | | | | | | | | |  |
| (Intercept) |  | 0.8052 | 0.8020 – 0.8083 | 0.00 | **<.001** |  | 0.8137 | 0.8112 – 0.8163 | 0.00 | **<.001** | |
| Trials (linear) |  | 0.0881 | 0.0822 – 0.0941 | 0.00 | **<.001** |  | 0.0866 | 0.0822 – 0.0910 | 0.00 | **.001** | |
| Trials (quadratic) |  | -0.0483 | -0.0523 – -0.0443 | 0.00 | **<.001** |  | -0.0493 | -0.0525 – -0.0460 | 0.00 | **.001** | |
| **Random Parts** | | | | | | | | | | |  |
| N_reset_session:user_ |  | 39230 | | | |  | 72741 | | | | |
| N_user_ |  | 5566 | | | |  | 8544 | | | | |
| Observations |  | 738946 | | | |  | 1354597 | | | | |
| R^2^ / Ω_0_^2^ |  | .402 / .395 | | | |  | .407 / .401 | | | | |

**Accuracy as a function of trial for samples 1 and 2 – Nested within session within user**

Notes: This analysis is the same as reported in S2 Table, with the exception that trials are now nested within session within user, instead of just within user as before.
